# Access to essential and innovative anti-cancer medicines: a longitudinal study in Nanjing, China

**DOI:** 10.1186/s12913-024-11285-5

**Published:** 2024-07-11

**Authors:** Zhaoliu Cao, Lili Wang, Rui Ma, Yun Hu, Baiyi Bao, Xiaohua Liu, Mengyuan Li, Xiao Wang, Pingyu Liu, Xin Li

**Affiliations:** 1 School of Pharmacy, Nanjing Medical University, Nanjing, Jiangsu 211166 China; 2 Nanjing City Qixia District Hospital, Nanjing, Jiangsu 210046 China; 3 Essential Medicine Division of Qixia District Health Commission of Nanjing City, Nanjing, Jiangsu 210046 China; 4 Department of Pharmacy, Jiangsu Province Hospital, the First Affiliated Hospital with Nanjing Medical University, Nanjing, Jiangsu 210029 China; 5 Department of Pharmacy, the Second Affiliated Hospital of Nanjing Medical University, Nanjing, Jiangsu 210011 China; 6 School of Health Policy and Management, Nanjing Medical University, Nanjing, Jiangsu 211166 China; 7 Center for Global Health, School of Public Health, Nanjing Medical University, Nanjing, Jiangsu 211166 China

**Keywords:** Anti-cancer medicines, Price, Availability, Affordability

## Abstract

**Purpose:**

To evaluate the availability, cost, affordability of anti-cancer medicines in Nanjing, Jiangsu.

**Methods:**

A longitudinal tracking investigation study was performed to collect information about 24 essential anti-cancer medicines (EAMs) and 17 innovative anti-cancer medicines (IAMs) in 26 healthcare institutions in Nanjing from 2016 to 2020. The availability, cost, drug utilization and affordability of EAMs and IAMs were investigated.

**Results:**

The availability of EAMs showed no significant changes in Nanjing, but the availability of IAMs showed a significant increase in 2018 and 2019 and tended to stabilize in 2020. For EAMs, the DDDc(Defined Daily Dose cost) of LPGs (Lowest-Priced Generics) showed no significant changes, and the DDDc of OBs (Originator Brands) and IAMs significantly decreased. The DDDs(Defined Daily Doses) of EAMs (LPGs) showed a decreasing trend since 2016 and rose again in 2019. Overall, the DDDs of EAMs (LPGs) decreased by 25.18% between 2016 and 2020, but the proportion selected for clinical treatment remained at 67.35% in 2020. The DDDs of EAMs (OBs) and IAMs both showed an increasing trend year by year, with a proportional increase of 207.72% and 652.68%, respectively; but the proportion selected for clinical treatment was only 16.09% and 16.56% respectively in 2020. EAMs (LPGs) had good affordability for urban residents but poor affordability for rural residents; the affordability of EAMs (OBs) and IAMs was poor for both urban and rural residents.

**Conclusions:**

There were no significant changes in the availability and cost of EAMs (LPGs), whose lower prices showed better affordability. Although their relative change in drug utilization showed a decreasing trend, they still dominated clinical treatment. Driven by the national drug price negotiation (NDPN) policy, the availability of IAMs was on the rise. It is necessary to further develop and strengthen policies for essential medicines procurement assessment to improve the accessibility of EAMs.

**Supplementary Information:**

The online version contains supplementary material available at 10.1186/s12913-024-11285-5.

## Introduction

In the world, cancer is the most significant obstacle to the increase in life expectancy due to its life-threatening nature. According to the data released by the International Agency for Research on Cancer (IARC), an estimated 19.3 million new cancer cases and 10.0 million cancer deaths were reported around the world in 2020, where China accounted for 23.7% of new cases and 30% of deaths, ranking the first in the world [1]. IARC predicted that there would be about 28.4 million new cancer cases worldwide by 2040, and the increase may be even higher in low-and middle-income countries [2].

The availability and affordability of medicines are of great importance for successfully implementing cancer screening, prevention, and treatment plans, particularly in developing countries [3]. However, the high price of innovative anti-cancer medicines, the imperfection of the supply system, and the poor quality of treatment greatly limit its availability and affordability [4]. The high out-of-pocket cost of health care is a major challenge for the healthcare system, while the unaffordable drug price is the most important reason why patients abandon treatment. Globally, cancer patients usually lack access to the medicines they need. In most low- and middle-income countries (LMICs), the high cost and poor availability of anti-cancer drugs are great obstacles to access. The patients’ monthly medicine expenditure often exceeds annual income [5]. For instance, a study conducted to evaluate the availability of anti-cancer medicines in Pakistan showed that the availability of anti-cancer medicines in public hospitals was 43% [6]. A Mexican study showed that the mean availability of anti-cancer medicines in public hospitals and private pharmacies was 61.2% and 67.5%, respectively [7]. Even in high-income countries, these high prices have begun to trigger a series of financial problems. For instance, the average drug cost of cancer treatment has grown 10-fold in the last 20 years in the UK [5].

China is the largest developing country in the world, and its per-capita GDP is much lower than that of most developed countries. Access to effective, cost-effective, and quality anti-cancer medicines is essential to improve health outcomes for cancer patients. To fulfill the priority health needs of populations, the Chinese health authority attaches great importance to the establishment and implementation of the National Essential Medicine System [8]. For nearly two decades, China has consistently promoted and effectively implemented a hierarchical medical system, as well as Two-way referral policies. Consequently, complex and severe illnesses such as cancer are predominantly managed in urban tertiary hospitals, with corresponding drug provisions. Cancer disease is a class of diseases characterized by significant harm, rapid progression, and high mortality rates, the majority of cancer patients are currently treated in tertiary hospitals across provinces and cities nationwide. Currently, in clinical practice, the treatment plans for cancers are required to adhere to the unified treatment guidelines published by national and international organizations. Therefore, the drug treatment regimens for the same disease are essentially consistent across regions. In summary, the data from the Nanjing region can largely reflect the situation in most regions of the country. Moreover, Nanjing is a typical city in Eastern China. Therefore, this study can also provide a demonstration of establishing a mechanism to improve market access to high-quality IAMs for other developing countries.

Essential medicines, which meet basic medical and health needs, have appropriate dosage forms, reasonable prices, can ensure supply, and are equitably accessible to the public [9]. In 2009, the National Essential Medicines List (NEML) was issued by the National Health Commission of China. In 2018, the NEML was updated and the growth rate of anti-cancer medicines was increased by 35%, which could greatly alleviate the burden of cancer treatment. Innovative anti-cancer medicines, which included small molecular targeted drugs and monoclonal antibodies, were introduced in the early 2000s and widely used in cancer therapy. Since 2016, more than 50 IAMs have been authorized in China. In the year of 2018, six small molecular targeted medicines were included in the NEML. Until 2022, IAMs have been included on the National Reimbursement Drug List. However, IAMs, authorized for use in China, did not deliver meaningful clinical benefits as expected and are more expensive than traditional chemotherapy drugs [10].

With the increasing incidence and mortality of cancer in China, improving access to anti-cancer medicines is a crucial part of health policy for cancer therapy and prevention. However, only a few studies have focused on the accessibility of anti-cancer medicines, especially for innovative medicines in China. A previous study surveyed the availability of 32 EAMs in the Hubei Province of China and found that on average availability of OBs in tertiary and secondary hospitals was 13.7% and 6.67%, respectively, whereas that of LPGs was relatively higher, at 62.83% and 42.92%, respectively [11]. In addition, a study aimed to evaluate the change in accessibility of EAMs from 2015 to 2018 in Anhui, China, all IAMs are unaffordable for urban and rural residents, but their affordability was on the rise [12].

Another national study focusing on procurement data found that the procurement of three out of 10 EAMs decreased, whereas the procurement of 10 targeted cancer medicines increased between 2015–2020 [13]. A study on EAMs for children showed that the availability of OBs and LPGs was 2.6% and 18.5% [14]. Our previous study focusing on 40 anti-cancer medicines in Jiangsu Province found that from 2012 to 2016 there was a significant decrease in the mean availability of LPGs (from 36.29 to 32.67%) and OBs (from 7.79–5.71%) [15].

However, most existing studies have three major drawbacks. (i) Only a limited number of IAMs were examined, especially for the data on availability and affordability. (ii) Little has been studied on the accessibility of anti-cancer medicines in eastern China since 2016, where the incidence of cancer diseases was the highest among different regions during 2015–2020 [12], with more aging population. (iii) The comparison between essential medicines and innovative medicines for cancer diseases still awaits further investigation. This study aims to measure access to essential and innovative anti-cancer medicines by the longitudinal survey data in a sample of public hospitals from 2016 to 2020 in Nanjing, Eastern China from 4 aspects as follows: availability, cost, drug utilization, and affordability.

## Methods

### Study design

We conducted a longitudinal study of medicine accessibility based on procurement data of healthcare institutions in Nanjing from 2016 to 2020, availability, cost, drug utilization and affordability were selected as the four analytical indicators. This study selected DDDc (defined daily dose cost) as the price index. Additionally, considering the characteristics of longitudinal tracking data in this study, we also added the DDDs (Defined Daily Doses) to observe the changes in the utilization of anti-cancer medicines in clinical practice. These two adjusted indicators have been used in previous studies [12, 13, 16]. The interpretation and calculation methods of the four indicators are shown in the data analysis section.

### Study target

Jiangsu Province is located on the eastern coast of mainland China, bordering Shandong, Shanghai, Zhejiang, and Anhui, with about 80 million residents. Jiangsu Province is one of the most economically advanced provinces in China, with a per capita GDP that has ranked first in China since 2009 [17].

There are 13 prefecture-level cities in Jiangsu Province, of which Nanjing is the capital city. Nanjing is one of the regional medical centers in the Yangtze River Delta region which has abundant medical resources, with 69 secondary hospitals and 37 tertiary hospitals [18], providing health services to residents of 1 municipality and 3 provinces (Shanghai Municipality, Zhejiang Province, Anhui Province, and Jiangsu Province).

In this study, we conducted a longitudinal study to analyze access to anti-cancer medicines in 26 secondary and higher healthcare institutions in Nanjing from 2016 to 2020 (Table S1). In China, only secondary and tertiary hospitals can provide specialized cancer therapy services for cancer patients. Few cancer inpatients admitted to the primary hospitals in Nanjing. In other words, the procurement data of secondary and tertiary hospitals are suitable for this study. Therefore, in this study, all of the tertiary hospitals in Nanjing were included. Six secondary hospitals were selected by convenience sampling. All of primary hospitals were excluded.

### Selection of medicines

According to the oncologists’ suggestions and local health statistics, five malignancies with a high morbidity and fatality rate were considered in this study: lung cancer, esophageal cancer, gastric cancer, liver cancer, and colorectal cancer. Leukemia, brain cancer, lymphoma, bone cancer, and liver cancer are the five deadly cancers for children and adolescents under 14 years old in Jiangsu Province [19].

EAMs were selected if they met the following criteria: (i) an anti-cancer medicine was included on the NEML 2018; (ii) on the WHO EML 2019; and (iii) approved for market in China before 2015. Thus, 24 essential anti-cancer medicines were selected.

Meanwhile, based on the definition and classification of IAMs in the Guiding Principles of Clinical Application of Innovative anti-cancer Medicines (GPCAIAMs 2020) issued by the National Health Commission (NHC) in 2020, the IAMs were selected if they met the following criteria: (i) an anti-cancer medicine was based on classification method by GPCAIAMs 2020. (ii) an anti-cancer medicine was included on the list of the GPCAIAMs 2020. (iii) If they are essential medicines at the same time, they will be listed as essential medicines for evaluation. Thus, 17 clinically commonly used and representative IAMs were selected and divided into three categories: 2 types of monoclonal antibodies, 11 types of targeted drugs, and 4 types of other targeted drugs. Tables 1 and 2 show the basic information of essential and innovative anti-cancer medicines, respectively. 41 medicines were surveyed from 2016 to 2020, selected from the commonly-used medicines in Nanjing, Jiangsu. For EAMs, the data was collected for two products: OBs and LPGs, only OBs marketed in China were analyzed. Due to the lack of generic drugs, only OBs data was collected for IAMs. Of the 41 medicines surveyed, 7 were for the treatment of lung cancer, 11 were for the treatment of Leukemia, 8 were for the treatment of Breast cancer and cervical cancer, 5 were for the treatment of gastrointestinal neoplasms and 10 were for other anti-cancer medicines or supportive medicines of cancer therapy.

**Table 1:** Basic information of essential anti-cancer medicines

Drug name	Dosage form	Strength	DDD (mg)	Main indication	NEML	WHO/ EML	Medical insurance medicine	Drug approval number of OBs	Drug approval number of LPGs
Cyclophosphamide	inj	200 mg	92	Leukemia	YES	YES	YES	YES	YES
Ifosfamide	inj	1 g	140	Testicular carcinoma	YES	YES	YES	YES	YES
Methotrexate	TAB-CAP	2.5 mg	2	Leukemia	YES	YES	YES	NO	YES
Mercaptopurine	TAB-CAP	50 mg	147	Leukemia	YES	YES	YES	NO	YES
Cytarabine	inj	100 mg	41	Leukemia	YES	YES	YES	YES	YES
Hydroxyurea	TAB-CAP	500 mg	800	head and neck cancer	YES	YES	YES	NO	YES
Fluorouracil	inj	250 mg	1050	Breast Cancer	YES	YES	YES	NO	YES
Gemcitabine	inj	200 mg	182	NSCLC	YES	YES	YES	YES	YES
Etoposide	inj	20 mg/ml	68	NSCLC	YES	YES	YES	NO	YES
Daunorubicin	inj	20 mg	61	Leukemia	YES	YES	YES	NO	YES
Vincristine	inj	1 mg	0.29	Leukemia	YES	YES	YES	NO	YES
Paclitaxel	inj	100 mg	14	Ovarian cancer	YES	YES	YES	YES	YES
Cisplatin	inj	10 mg	5	Ovarian cancer	YES	YES	YES	NO	YES
Oxaliplatin	inj	50 mg	11	Colorectal Cancer	YES	YES	YES	YES	YES
Carboplatin	inj	150 mg	24	Ovarian cancer	YES	YES	YES	YES	YES
ArsenicTrioxide	inj	5 mg	10	Leukemia	YES	YES	YES	NO	YES
Asparaginase	inj	10000IU	1700IU	Leukemia	YES	YES	YES	NO	YES
olinic acid calcium salt hydrate	inj	0.1 g	425	Adjuvant	YES	YES	YES	NO	YES
Capecitabine	TAB-CAP	500 mg	2833	Colorectal Cancer	YES	YES	YES	YES	YES
Tamoxifen	TAB-CAP	10 mg	20	Breast Cancer	YES	YES	YES	NO	YES
Mesna	inj	400 mg	1680	Adjuvant	YES	YES	YES	YES	YES
Imatinib	TAB-CAP	100 mg	400	Leukemia	YES	YES	YES	YES	YES
Rituximab	inj	100 mg	116	Non-Hodgkin’s lymphoma	YES	YES	YES	YES	YES
Trastuzumab	inj	0.44 g	20	Breast Cancer	YES	YES	YES	YES	YES

DDD, defined daily dose; NEML, National Essential Medicines Lists; WHO/EML, World Health Organization/Essential Medicines Lists; LPGs, lowest-price generics; OBs, originator brands; inj, injection; TAB, Tablet; CAP, Capsule; YES, on the list; NO, not on the list

**Table 2:** Basic information of innovative anti-cancer medicines

Drug name	Dosage form	Strength	DDD (mg)	NEML	WHO/ EML	Medical insurance medicine	Main Indication	Registration classification	Drug approval number of OBs	Drug approval number of LPGs
Bevacizumab	inj	0.1 g	50	NO	YES	YES	Colorectal cancer	Biological products 3.1	YES	YES
Nimotuzumab	inj	50 mg	14	NO	NO	YES	Nasopharyngeal carcinoma	Biological products 1	YES▲	NO
Dasatinib	TAB-CAP	50 mg	100	NO	YES	YES	Leukemia	Chemicals 5.1	YES	YES
Gefitinib	TAB-CAP	250 mg	250	YES	NO	YES	NSCLC	Chemicals 5.1	YES	YES
Lapatinib	TAB-CAP	0.25 g	1250	NO	NO	YES	Breast Cancer	Chemicals 5.1	YES	NO
Apatinib	TAB-CAP	0.25 g	1250	NO	NO	YES	Gastric Cancer	Chemicals 1	YES▲	NO
Crizotinib	TAB-CAP	250 mg	500	NO	NO	YES	NSCLC	Chemicals 5.1	YES	YES
Nilotinib	TAB-CAP	200 mg	600	NO	YES	YES	Leukemia	Chemicals 5.1	YES	YES
Sunitinib	TAB-CAP	12.5 mg	33	NO	NO	YES	Gastrointestinal Stromal Tumor	Chemicals 5.1	YES	YES
Icotinib	TAB-CAP	125 mg	125	YES	NO	YES	NSCLC	Chemicals 1	YES▲	NO
Erlotinib	TAB-CAP	150 mg	150	NO	YES	YES	NSCLC	Chemicals 5.1	YES	YES
Axitinib	TAB-CAP	5 mg	10	NO	NO	YES	Nephrocytoma	Chemicals 5.1	YES	YES
Sorafenib	TAB-CAP	0.2 g	800	NO	NO	YES	Nephrocytoma	Chemicals 5.1	YES	YES
Bortezomib	inj	3.5 mg	0.42	NO	YES	YES	Myeloma	Chemicals 5.1	YES	YES
Everolimus	TAB-CAP	5 mg	10	NO	YES	YES	Nephrocytoma	Chemicals 5.1	YES	YES
Chidamide	TAB-CAP	5 mg	9	NO	NO	NO	T-cell lymphoma	Chemicals 1	YES▲	NO
Rh-endostatin	inj	15 mg	9	NO	NO	NO	NSCLC	Biological products 1	YES▲	NO

DDD, defined daily dose; NEML, National Essential Medicines Lists; WHO/EML, World Health Organization/Essential Medicines Lists; LPGs, lowest-price generics; OBs, originator brands; inj, injection; TAB, Tablet; CAP, Capsule; YES, on the list; NO, not on the list; Biological products 3.1, The first marketing approval application for therapeutic biological products which have been approved for marketing abroad; Biological products 1, The first marketing approval application for the therapeutic biological products which were not approved for marketing worldwide; Chemicals 5.1, The first marketing approval application for the original brands and improved drugs that have been approved for marketing abroad. The drugs that are improved should have obvious clinical advantages. Chemicals 1, The first marketing approval application for Innovative drugs that have not been approved at home and abroad; NSCLC, Non-small cell lung cancer.▲: The original research conducted in China

We collected the monthly procurement data of 41 anti-cancer medicines in Nanjing from 2016 to 2020 from the database of Jiangsu Institute of Medicine Information. The data items included: generic name (including dosage form), trade name, pharmaceutical manufacturer, specification, unit price, name of purchasing unit, and date of purchase. A total of 26 healthcare institutions were included in the study, accounting for 24.53% of secondary and tertiary hospitals in Nanjing, including 6 secondary hospitals (8.7%) and 20 tertiary hospitals or tumor specialty hospitals (54.05%). The included sample hospitals were all required to set departments or disciplines for oncology diagnosis and treatment.

### Data analysis

This study was analyzed by 4 indicators: availability, drug utilization, cost, and affordability.

The availability of medicines was calculated as the proportion of all the surveyed institutions where a specific drug can be found.

$$Availability = {\matrix{the\;number\;of\;institutions \hfill \cr \;that\;can\;provide\;the\;drug \hfill \cr} \over \matrix{the\;total\;number\;of\; \hfill \cr research\;institutions \hfill \cr} } \times 100\%$$

The following criteria were used to evaluate the availability of anti-cancer medicines [20, 21]. 0% indicates that it is not available in any hospital surveyed; < 30% was very low availability; 30–49% indicates low availability; 50–80% had fairly high availability; > 80% indicates high availability. WHO has set 80% as the threshold for the target for availability [22]. In this study, we assume that a drug is available in the healthcare institution if it has sales records in any month of the year, the median and interquartile range of the availability of EAMs (LPGs and OBs) and IAMs were calculated year by year, as well as the median and Interquartile Range (IQR) of the difference in availability between adjacent years. Wilcoxon’s rank-sum test was used to test whether there were significant differences in the availability of adjacent years, and *P* < 0.05 was used to show a significant difference.

Based on the WHO Collaborative Center for Drug Statistics Methodology, the cost was expressed as the defined daily dose cost (DDDc) of each anti-cancer medicine surveyed in this study, which was calculated based on the DDD. We examined the significance of the temporal trend in the median DDDc of medicines by using Wilcoxon’s rank-sum test. Because anti-cancer medicines are generally calculated according to a course of treatment, the total cost of a course of treatment was apportioned in this study to each day, rather than the cost spent on the actual days of use, to be consistent with the concept of qualified daily cost [23]. A higher DDDc indicated a more expensive medicine. DDDc is calculated as follows.

$$\:DDDc=\frac{total\:cost\:of\:a\:drug\:use}{defined\:daily\:doses\:\left(DDDs\right)}$$

Defined Daily Doses (DDDs) are cumulative Defined Daily Doses (DDD) that provide a fixed unit of measurement independent of currency type, price, packaging, and specification, etc. DDDs are used to measure trends in drug utilization, with larger values representing a greater trend in the selection of a drug [24]. The DDDs were calculated using the following formula.

$$\:DDDs=\frac{Total\:doses\:of\:drug\:use}{defined\:daily\:dose\left(DDD\right)}$$

Due to highly individualized treatment schedules, WHO doesn’t assign DDD for topical medications, serums, vaccines, anti-cancer medicines, allergen extracts, general and local anesthetics, and contrast agents [25]. We used the daily dose in the medicine’s product information for the main treatments as a reference, due to the absence of a DDD for an anti-cancer medicine [13].

In this study, affordability is an indicator that assesses the ability of patients to afford drugs within a given economic area. The affordability is determined by the ratio of the treatment cycle cost to per capita disposable income, which is calculated by the wage of the lowest-paid unskilled government worker (LPGW). It should be noted that affordability may vary across regions with different economic levels, or even among patients covered by different types of medical insurance within the same economic level area. Considering the significant differences between urban and rural areas in China and the lack of data on LPGW, we adopted the per capita disposable income in Nanjing as a reference in this study [26, 27]. Information on the per capita disposable income of urban and rural residents in Nanjing in 2016 and 2020 was collected from the Nanjing Statistical Yearbook (2021 edition) [28].

Considering local health insurance regulations and reimbursement policies, the out-of-pocket ratio of the national essential medical insurance program for urban employed in Nanjing was set at 15% and 30% for rural residents [29]. Treatment courses were calculated as 7-day doses for acute diseases and 30-day doses for chronic diseases [30]. Out-of-Pocket health expenditures were considered affordable if they were less than or equal to the per capita disposable income (daily wage) in Nanjing, and unaffordable if the opposite was true. The formula for calculating affordability is as follows.

$$Affordability = {\matrix{total\;cost\;of\;30\;days\;of\;drug\;use \times \; \hfill \cr the\;ratio\;of\;\;out - of - pocket \hfill \cr} \over \matrix{per\;capita\;disposable \hfill \cr \;income(daily\;wage) \hfill \cr} }$$

To identify the comprehensive situation, a four-quadrant diagram was performed to display the results of the availability and affordability of the medicines simultaneously. The affordability value of the medicines for patients in rural or urban areas was shown on the Y-axis, while the availability value was depicted on the X-axis. In the scattered plot, the lower right quadrant (IV) showed the medicines with low affordability and low availability, the lower left quadrant (III) showed the medicines with low affordability and high availability, the upper right quadrant (II) showed the medicines with low affordability and high availability, and the upper left quadrant (I) showed the medicines with low affordability and high availability.

Data were analyzed using SPSS (version 26.0; SPSS, Inc., Chicago, IL, USA) and Matlab (version 2018; MathWorks, Natick, MA, USA).

## Results

### Availability

The availability of 41 anti-cancer medicines is shown in Table 3, stratified by EAMs and IAMs. For EAMs (LPGs and OBs), there were no significant changes in surveyed 26 healthcare institutions from 2016 to 2020 in Nanjing. However, IAMs has a significant increase from 2017 to 2019 (*P*<0.01), and then stabilized afterward.

Besides, Fig. 1 shows the variation in the distribution of medicine availability. For LPGs of EAMs, the largest proportion of medicine availability was<30% and only the availability of Oxaliplatin was >80% per year. For OBs of EAMs, the largest proportion of medicine availability ranged from 30 to 49%, only the availability of Mesna was <30%. For IAMs, the largest proportion of medicine availability was <30% from 2016 to 2018, whereas the proportion ranged from 30 to 49% from 2019 onwards, only Bevacizumab availability was>80% in 2018 and 2019. Some medicines (LPGs of EAMs) were difficult to access in 26 hospitals, with Mercaptopurine and Carboplatin being available at <10%.

**Table 3:** Median availability of 41 anti-cancer medicines from 2016 to 2020 in Nanjing (%)

Year	EAMs				IAMs	
	LPGs		OBs			
	Availability (IQR)	Median change in availability (IQR)	Availability (IQR)	Median change in availability (IQR)	Availability (IQR)	Median change in availability (IQR)
2016	36.53(46.15)	-	42.00(21.00)	-	19.00(15.00)	-
2017	30.77(69.23)	0.00(11.54)	42.00(31.00)	0.00(5.77)	15.38(23.00)	0.00(7.69)
2018	34.60(53.85)	0.00(7.69)	40.38(39.99)	1.92(7.69)	27.00(30.00)	3.85(11.54)**
2019	34.60(53.85)	1.92(11.54)	42.31(40.00)	3.85(9.62)	38.00(27.00)	3.85(3.85)**
2020	40.3(65.38)	0.00(7.69)	40.38(24.99)	-3.85(9.62)	38.00(22.99)	-3.85(7.79)

EAMs, Essential anti-cancer medicines; IAMs, Innovative anti-cancer medicines; LPGs, lowest-price generics; OBs, originator brands; IQR: interquartile range; Median change in availability, median availability change of each medicine surveyed between adjacent years

### **P<0.01

**Fig. 1:**
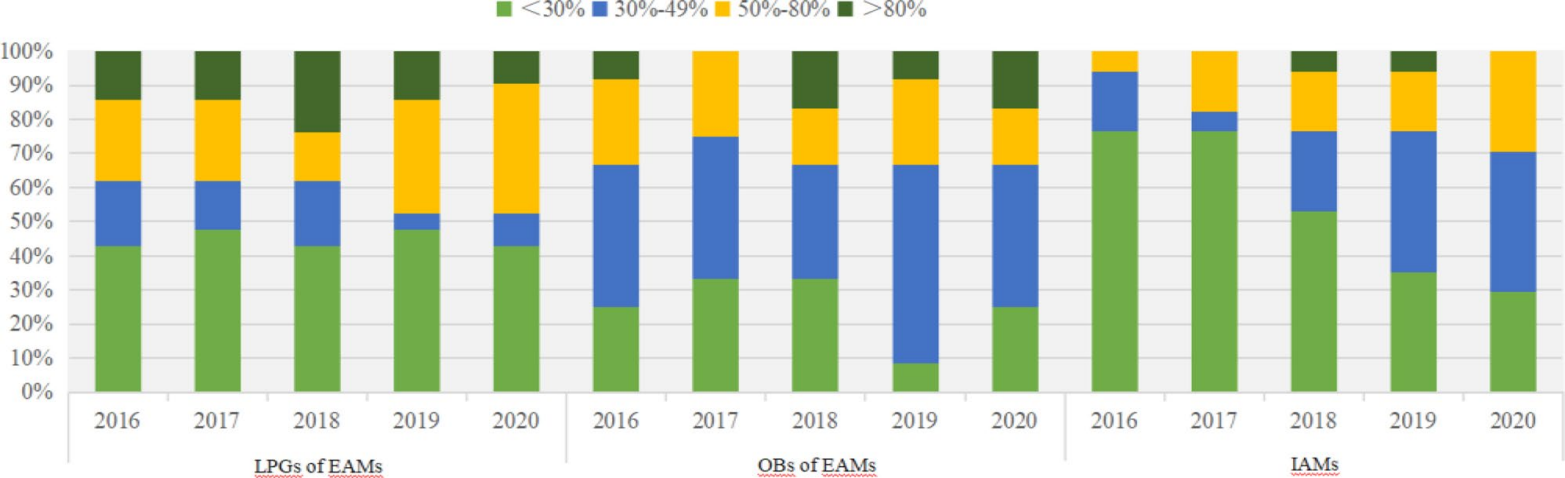
Availability of 41 anti-cancer medicines from 2016 to 2020 in Nanjing(%)

### Cost

The median DDDc of anti-cancer medicines surveyed in Nanjing from 2016 to 2020 is shown in Table 4. Of the sampled outlets, prices were available for 12 OBs of EAMs, 19 LPGs of EAMs, and 17 IAMs. The largest DDDc of EAMs (LPGs), EAMs (OBs), and IAMs are Paclitaxel, Rituximab, and Bevacizumab, respectively. The results of Wilcoxon’s rank-sum test showed that the DDDc of EAMs (OBs) remained stable during 2016–2017, whereas there was a significant decline from 2018 to 2020 (*P*<0.05). The DDDc of EAMs (LPGs) was fairly steady during the survey period. Furthermore, the median DDDc of IAMs has declined significantly per year.

**Table 4:** Median DDDc of medicines surveyed from 2016 to 2020 in Nanjing

Type		Year	DDDc(IQR)	Median of product-specific change (IQR)
EAMs	LPGs	2016	12.13(87.24)	-
		2017	13.68(87.09)	-0.91(2.73)
		2018	15.19(62.10)	0(3.84)
		2019	13.20(50.01)	-0.099(6.65)
		2020	11.69(55.19)	-0.29(6.37)
	OBs	2016	193.05(766.57)	-
		2017	192.99(518.24)	0.00(2.78)
		2018	180.56(475.07)	-12.43 (60.61) *
		2019	165.46(416.01)	-14.93 (55.22) **
		2020	155.78(315.80)	-1.59 (11.49) *
IAMs		2016	900.00(733.35)	-
		2017	697.2(613.39)	-118.12(270.69)**
		2018	484.01(412.85)	-92.02(208.47)**
		2019	409.20(361.64)	-11.40(27.87)**
		2020	358.26(154.00)	-27.12(118.44)*

EAMs, Essential anti-cancer medicines; IAMs, Innovative anti-cancer medicines; LPGs, lowest-price generics; OBs, originator brands; IQR: interquartile range; Median of product-specific change, the median of daily cost change of each medicine surveyed between adjacent years

*P<0.05

**P<0.01

### Drug utilization

**Fig. 2:**
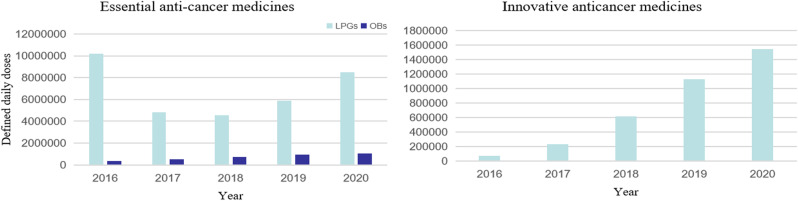
Changes in DDDs of anti-cancer medicines from 2016 to 2020 in Nanjing

Overall, the DDDs of EAMs (LPGs and OBs) were much higher than IAMs in all years (Fig. 2), especially for EAMs (LPGs). Table 5 summarizes the changes in mean DDDs of anti-cancer medicines in 2016 and 2020 in Nanjing. The mean DDDs of IAMs increased each year from 12088.01 in 2016 to 90984.12 in 2020. For LPGs of EAMs, 494575.43 DDDs were used in 2016, While the mean DDDs began to decline in 2017, it increased again in 2019 to 267361.80 DDDs. The proportion of EAMs (LPGs) and IAMs in clinical diagnosis and treatment was 92.38% and 2.26% in 2016, and 67.35% and 16.56% in 2020, respectively. Significantly, the mean DDDs of EAMs (OBs) had increased over time. According to available procurement data, it was found that among the 22 surveyed EAMs (LPGs), 50% of the drugs experienced an increase in DDDs. Among the 12 surveyed EAMs (OBs),10 showed an increase in DDDs, representing 83.33%. Notably, all 17 IAMs have shown an increase in their DDDs(Table S2 and Table S3).

**Table 5:** Changes in mean DDDs of anti-cancer medicines in 2016 and 2020

Type		Mean DDDs/Percent (2016)	Mean DDDs/Percent(2020)	Absolute change	Relative change (%)
EAMs	LPGs	494575.43/92.38%	370041.86/67.35%	-124533.57	-25.18%
	OBs	28735.16/5.36%	88425.19/16.09%	59690.03	207.72%
IAMs		12088.01/2.26%	90984.12/16.56%	78896.11	652.68%

DDDs, Defined daily doses; LPGs, lowest-price generics; OBs, originator brands; EAMs, essential anti-cancer medicines; IAMs: innovative anti-cancer medicines

### Affordability

Because of the lack of continuous data for some anti-cancer medicines, we calculated the affordability of 12 EAMs (OBs), 19 EAMs (LPGs), and 17 IAMs for urban and rural residents from 2016 to 2020 (Tables 6 and 7). Generally, the affordability of each medicine showed improvement for urban and rural residents in Nanjing over time. In 2016, the most affordable standard treatment for residents was the treatment of NSCLC (Non-small cell lung cancer) with IAMs Crizotinib (57.41 for urban residents and 271.35 for rural residents), which declined to 12.64 for urban residents and 57.67 for rural residents in 2020. Except for the affordability of EAMs (LPGs) in 2017 and 2019 for urban residents, there was a significant improvement for rural residents and urban residents (*P*<0.01). All IAMs are unaffordable for both urban and rural residents; the EAMs (OBs), except for Cyclophosphamide, Gemcitabine, and Carboplatin, are unaffordable for urban residents, and all except for Cyclophosphamide are unaffordable for rural residents. However, the affordability of these drugs has been continuously improving from 2016 to 2020, and with a significant difference between adjacent years.

**Table 6:** Median affordability of essential anti-cancer medicines from 2016 to 2020 in Nanjing

Type	Year	Urban		Rural		P
		Affordability (IQR)	Median change in Affordability (IQR)	Affordability (IQR)	Median change in Affordability (IQR)	
LPGs	2016	0.47(2.21)	-	2.22(10.44)	-	0.000
	2017	0.54(1.88)	-0.04(0.27)	2.58(8.84)	-0.19(1.30)*	0.000
	2018	0.57(1.59)	-0.09(0.52)**	2.65(7.42)	-0.46(2.53)**	0.000
	2019	0.59(1.52)	-0.03(0.19)	2.73(7.10)	-0.14(0.99)*	0.000
	2020	0.38(1.25)	-0.05(0.37)*	1.68(5.67)	-0.26(1.90)*	0.000
OBs	2016	12.70(20.42)	-	60.03(96.50)	-	0.003
	2017	11.64(18.79)	-1.06(1.62)**	54.90(88.65)	-5.13(7.85)**	0.003
	2018	9.79(15.18)	-1.86(3.57)**	45.95(71.28)	-8.95(17.16)**	0.003
	2019	7.90(8.93)	-1.64(5.19)**	36.80(41.59)	-8.04(25.49)**	0.003
	2020	7.53(8.50)	-0.40(0.85)**	34.34(38.81)	-2.66(4.48)**	0.000

P-value reported in the table is used to reflect the difference of essential anti-cancer median affordability between urban and rural residents; LPGs, lowest-price generics; OBs, originator brands; Median change in Affordability, the median of affordability change of each medicine surveyed between adjacent years; IQR: interquartile range

*P<0.05

**P<0.01

**Table 7:** Median affordability of innovative anti-cancer medicines from 2016 to 2020 in Nanjing

Type	Year	Urban		Rural		P
		Affordability (IQR)	Median change in Affordability (IQR)	Affordability (IQR)	Median change in Affordability (IQR)	
IAMs	2016	28.27(25.62)	-	133.60(121.12)	-	0.000
	2017	13.65(9.64)	-1.15(14.98)**	64.37(45.44)	-5.58(70.83)**	0.000
	2018	11.63(7.68)	-1.02(1.67)**	54.62(36.06)	-5.02(7.83)**	0.000
	2019	10.56(6.89)	-0.91(0.62)**	49.21(32.10)	-4.70(3.18)**	0.000
	2020	7.94(3.99)	-2.77(2.22)**	36.23(18.18)	-13.69(9.67)**	0.000

P-value reported in the table is used to reflect the difference of innovatice anti-cancer median affordability between urban and rural residents; Median change in Affordability, the median of affordability change of each medicine surveyed between adjacent years; IQR: interquartile range; IAMs, Innovative anti-cancer medicines

**P<0.01

### Comprehensive analysis of affordability and availability

**Fig. 3:**
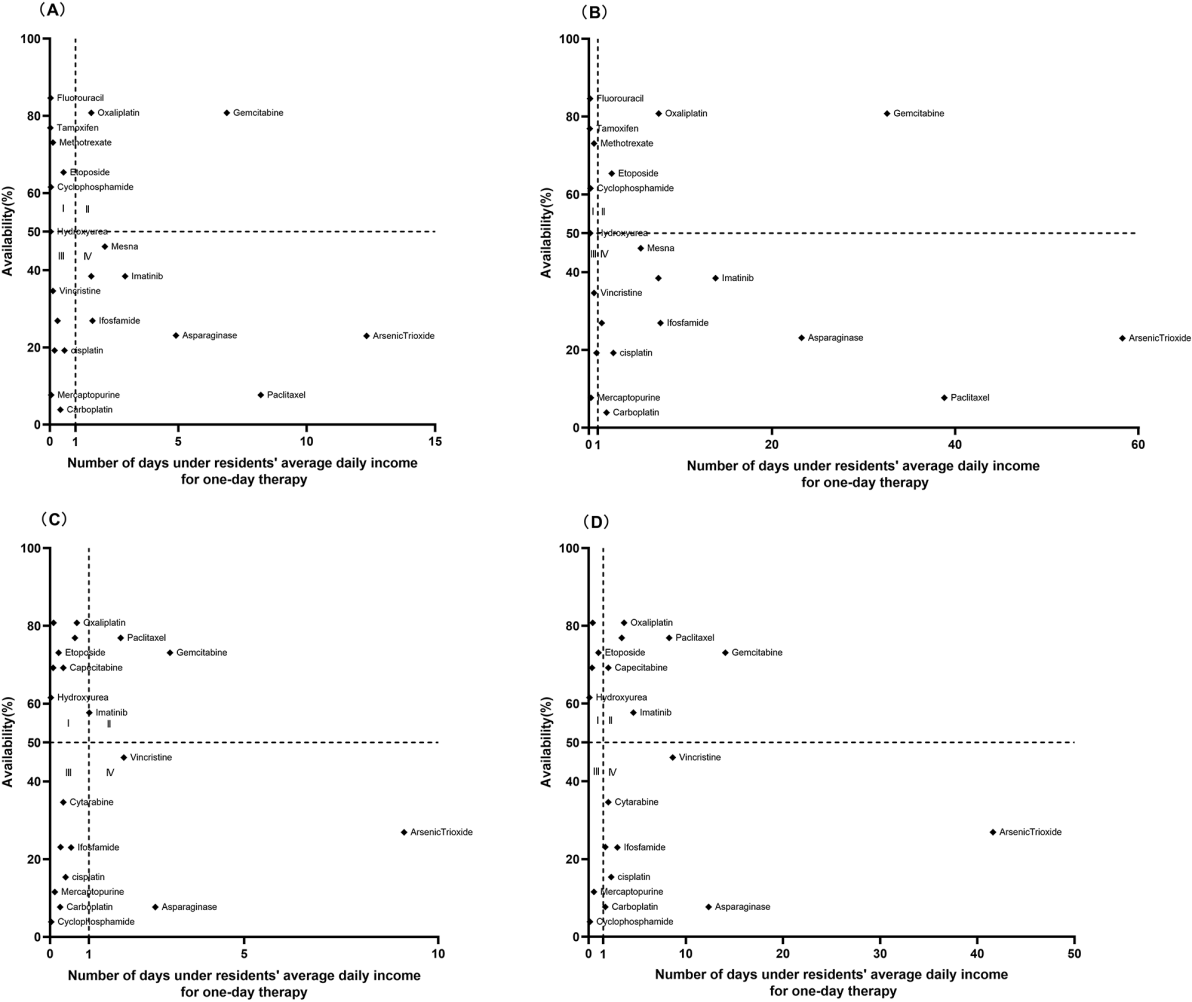
Comprehensive analysis of the EAMs availability and affordability in Nanjing in 2016 and 2020. (**A**) Comprehensive analysis of LPGs availability and affordability for urban residents in 2016. (**B**) Comprehensive analysis of LPGs availability and affordability for rural residents in 2016. (**C**) Comprehensive analysis of LPGs availability and affordability for urban residents in 2020. (**D**) Comprehensive analysis of LPGs availability and affordability for rural residents in 2020

**Fig. 4:**
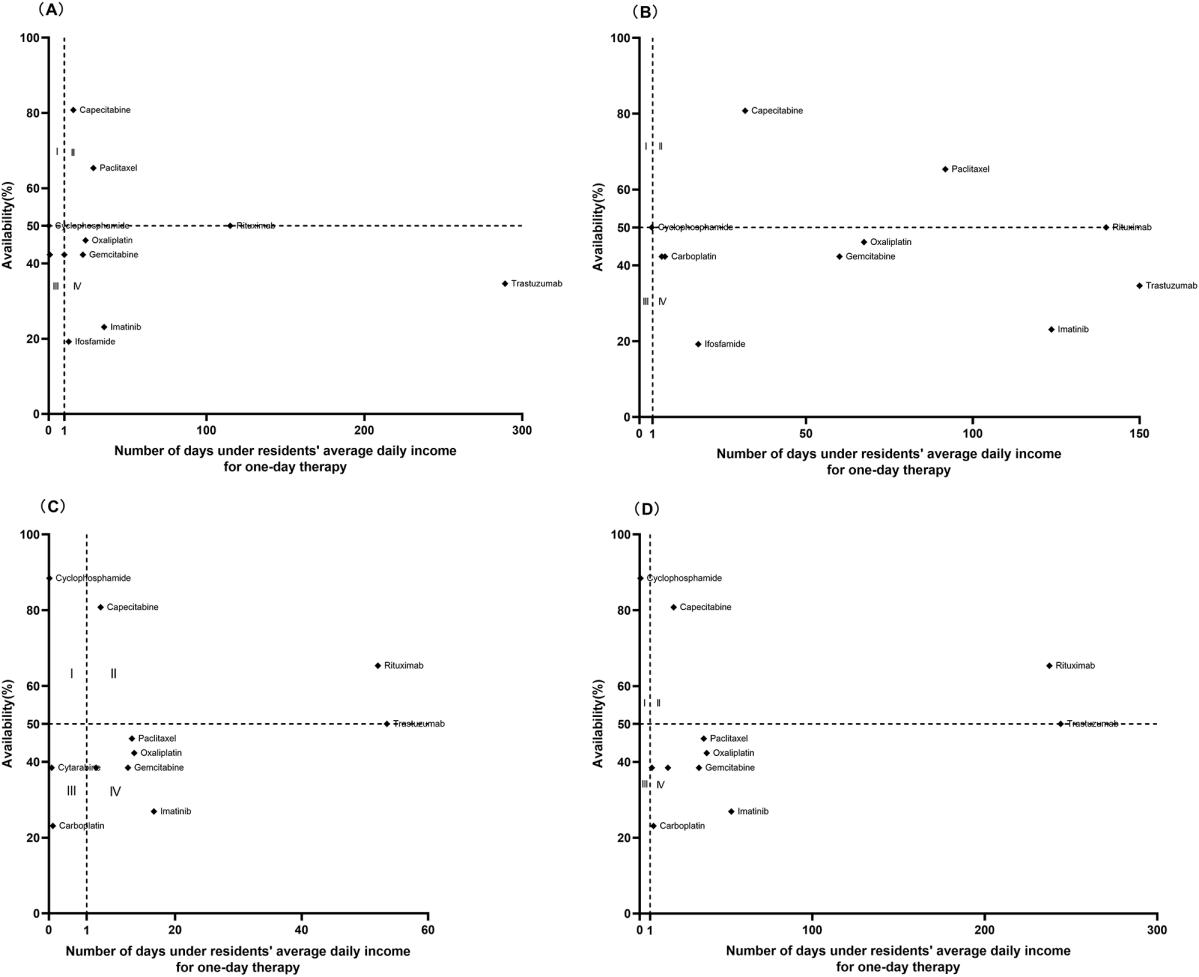
Comprehensive analysis of the EAMs availability and affordability in Nanjing in 2016 and 2020. (**A**) Comprehensive analysis of OBs availability and affordability for urban residents in 2016. (**B**) Comprehensive analysis of OBs availability and affordability for rural residents in 2016. (**C**) Comprehensive analysis of OBs availability and affordability for urban residents in 2020. (**D**) Comprehensive analysis of OBs availability and affordability for rural residents in 2020

**Fig. 5:**
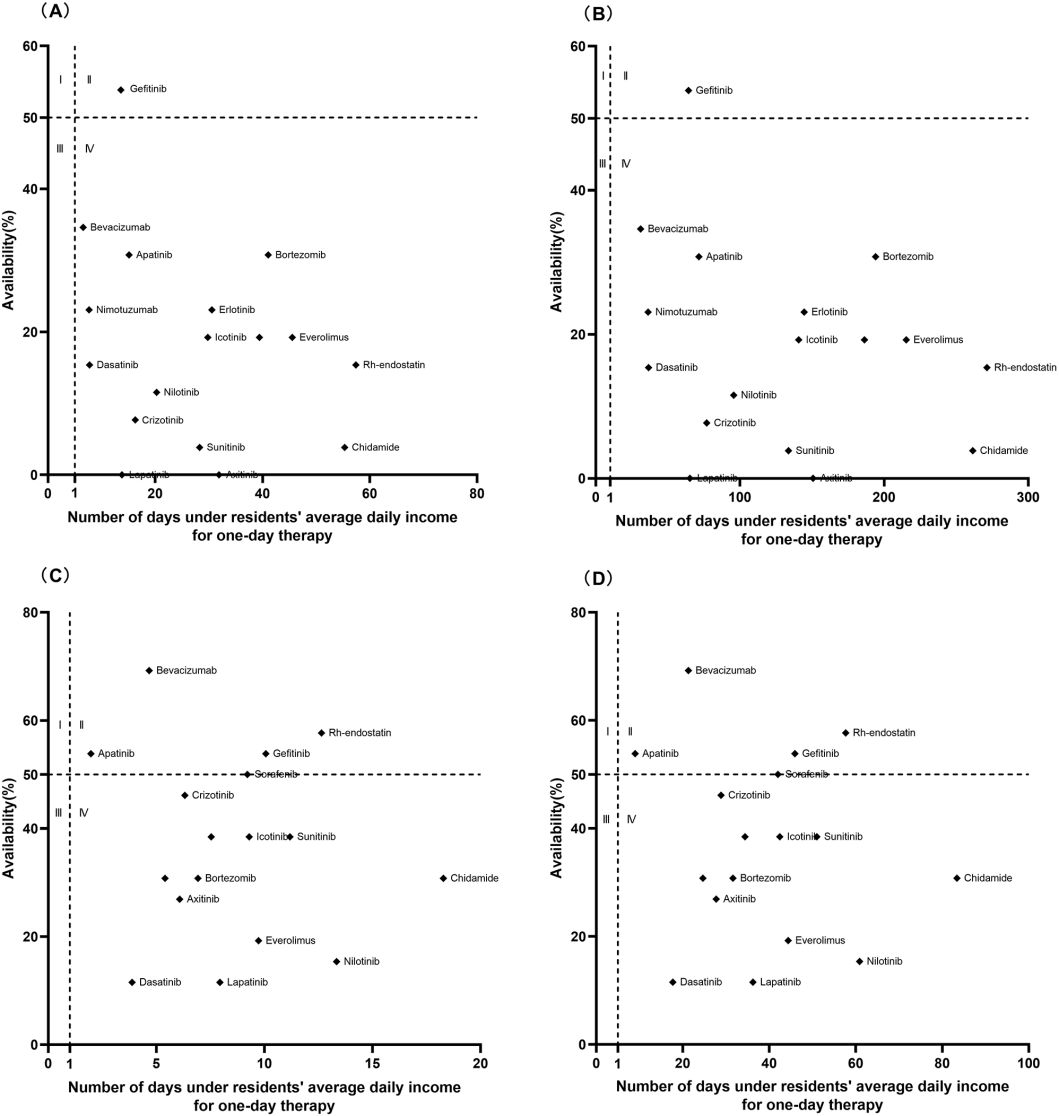
Comprehensive analysis of the IAMs availability and affordability in Nanjing in 2016 and 2020. (**A**) Comprehensive analysis of availability and affordability for urban residents in 2016. (**B**) Comprehensive analysis of availability and affordability for rural residents in 2016. (**C**) Comprehensive analysis of availability and affordability for urban residents in 2020. (**D**) Comprehensive analysis of availability and affordability for rural residents in 2020

A comprehensive analysis of affordability and availability for residents in 2016 and 2020 is shown in Figs. 3, 4 and 5. The graph was divided into four districts using the 50% medicine availability and the average daily income as criteria. The availability of most EAMs changed little but affordability improved over time. However, some LPGs of EAMs (Tamoxifen, Fluorouracil) showed the opposite situation, with improved availability and increased affordability of medication. The availability and affordability of IAMs had improved over time, with the availability of >50% of medicines rising from one to five, but rural and urban residents still could not afford IAMs, the least affordable standard treatment for residents was the treatment of Colorectal Cancer with Bevacizumab(4.67 for urban residents and 21.31 for rural residents) until 2020.

## Discussion

In this study, we adopted a longitudinal tracking survey method to conduct research and analysis on the accessibility of 41 anti-cancer medicines in 26 secondary and above hospitals in Nanjing from 2016 to 2020. The main research findings are as follows:

First, the availability of EAMs remained relatively stable during the study. This can be attributed to a series of policies implemented in recent years, which have resulted in a domestic production rate of 100% for anti-cancer medicines required for daily treatment. The supply of the 24 EAMs included in this study is relatively mature in the Chinese market, ensuring overall stability in their accessibility and effectively guaranteeing their availability for patients. The mean availability of EAMs (LPGs and OBs) and IAMs was low (<50%) during the study period. The results of this analysis are similar to those of a previous longitudinal tracking study on the availability of EAMs [15]. A cross-sectional study in Hubei Province, China showed that the availability of EAMs (LPGs) was 40.88%, while that of OBs was only 7.57% [11].

In this study, the mean availability of OBs was higher than that of the two studies above, which may be due to the exclusion of some essential medicines in the evaluation of the mean availability of OBs, such as Methotrexate, Tamoxifen, Hydroxyurea, etc., which do not have the approval of the original manufacturer in China, that is, they are not listed or have been withdrawn from the market in China.

The results of a cross-sectional survey of 82 countries showed that the availability of the top 20 anti-cancer medicines ranged from 9 to 54% in low-middle-income countries [31]. The mean availability of essential and non-essential anti-cancer medicines in hospitals in the Ghana region was 27% and 38%, respectively [32]. In Mexico, the situation appears to be better than in other regions, with the availability of EAMs in hospitals reaching 61.2%, particularly for drugs labeled “People’s Health Insurance” which have higher availability [6].

The relatively low availability of EAMs may be due to a variety of key factors, such as the lack of an incentive mechanism for the use of essential medicines and the negative impacts of the “drug zero markup” policy. For instance, at the end of 2015, the “drug zero mark-up” policy was fully promoted in public hospitals in Nanjing city, which reduced the original income of healthcare institutions. It may also be the reason for the low motivation for essential medicine. Although the government has reduced drug expenditure to meet the needs of the public, the support for public hospitals remains unchanged [33]. With the decline in revenue of public hospitals, the proportion of funds occupied by drugs has to be reduced to maintain the normal operation of the hospital, so it is difficult to maintain sufficient stocks of these life-saving drugs.

Second, although the availability of IAMs increased over the study period, we found that the EAMs had better coverage than IAMs. The higher availability of EAMs can be attributed to China’s national essential medicines system and the performance appraisal system for public hospitals. Since 2009, the Chinese central government had established a national essential system to meet the basic healthcare needs of the public [34]. In recent years, performance assessment systems had been established in the different levels of public hospitals in China. The implementation of the national essential medicines system had been incorporated into the performance assessment indicators. In order to achieve the assessment indicators, each local government set specific targets for different levels of public hospitals in essential medicines usage. For example, the secondary hospitals in Jiangsu province had set a target of at least 40% of all drugs sales amount and sales volume being for essential medicines [35].

On the other hand, the availability of IAMs has increased year by year in this study, but it is still low (<50%). In recent years, the development of IAMs has been continuous, especially since 2015, the speed of approval and listing of IAMs such as targeted anti-cancer medicines and monoclonal antibodies in China has been accelerating [36]. Another reason is the market distribution. A survey on the market distribution format of innovative anti-cancer medicines in Nanjing discovered that domestically produced anti-cancer medicines account for approximately 65% of all IAMs marketed in Nanjing, China [37]. Among the 17 IAMs included in this study, 5 IAMs are domestically developed in China, and 11 out of the 12 foreign-origin IAMs are produced in China. The localization plays a crucial role in improvements to the accessibility of anti-cancer medicines.In addition, its precise clinical effect and the national drug price negotiation (NDPN) policy implemented by China after 2016 have reduced the price of IAMs to enter the medical insurance catalog, which has improved the availability of IAMs in healthcare institutions [23, 38, 39]. The continuous research and development of IAMs may affect the availability of EAMs in healthcare institutions.

Third, we found that the DDDc of EAMs (LPGs) did not change significantly between adjacent years, while there was a significant decline of EAMs (OBs) between the adjacent years from 2018 to 2020. IAMs showed a downward trend year by year. The possible reason for the stable price of EAMs (LPGs) is that since 2016, China has always required a consistent evaluation of the quality and efficacy of generics to improve international competitiveness. The upgrade of the production process has improved the quality of generics, which has also led to higher production costs. Generally, LPGs are priced lower than OBs [40]. Therefore, it is a reasonable decision for consistent evaluation to lead to the increase of enterprise production costs and maintain the original price or increase appropriately.

Most EAMs (OBs) and IAMs are expensive, and the prices of these two types of drugs are generally high and unaffordable in many similar studies [13, 23, 38, 39]. The reason for the overall downward trend of the prices of the two types of drugs may be related to the implementation of the NDPN policy in China since 2016 [38]. From 2016 to 2020, 63 anti-cancer medicines were included in the NDPN catalog, 57 of which were chemical drugs, with an average price reduction of more than 44% [41]. Among the 17 IAMs included in this study, only Lapatinib failed to be renewed in 2019, and the other 16 IAMs were in the NDPN catalog.

Fourth, we found that the drug utilization of EAMs is higher than that of IAMs, in other words, essential medicines dominated in clinical diagnosis and treatment. During the study period, the total drug utilization of essential medicines showed a downward trend, but slowly increased after 2019, while the drug utilization of IAMs continued to increase, which is consistent with the results of a study on the utilization of anti-cancer medicines nationwide [13, 40]. The dominance of EAMs in cancer diagnosis and treatment can be attributed to the reimbursement policies of medical insurance and the prices of drugs. Of the 24 EAMs included in this study, 16 were reimbursed by medical insurance Class-A, with a reimbursement rate of 100%; while the 16 IAMs included in the NDPN catalog were all medical insurance Class-B, with a requirement of no more than 30% self-payment standard [42]. Therefore, the reimbursement policy for EAMs is better than that for IAMs. At the same time, the price of IAMs is much higher than that of EAMs [43], and its high price brings a great economic burden to patients, which to some extent affects patients’ choice of IAMs.

In addition, the relative change of DDDs of IAMs is much larger than that of EAMs. Although the reimbursement level of IAMs is lower than that of EAMs, the inclusion of IAMs in medical insurance can promote greater drug utilization [44]. In this study, the utilization of all IAMs has increased. This result is consistent with a published study, which showed that the sales of all types of anti-cancer medicines in China increased from 2007 to 2017 [45]. In contrast, our study showed that the utilization of some EAMs was declining.

In our analysis of IAMs, we found that the utilization of Erlotinib has increased significantly from 2016 to 2020, which may be related to the successful first NDPN of Erlotinib in 2016, with a price reduction of 54%, and its direct inclusion in the regular medical insurance catalog in 2017 [39]. The smallest increase in drug utilization was for Crizotinib, which is consistent with the results of a regional study in China [46]. Crizotinib was included in the NDPN in October 2018 and entered the Medical Insurance B-level catalog with a price reduction of approximately 23147.81-26041.29 dollars per year. The smallest increase in Crizotinib may be due to its limited effectiveness and drug resistance for brain metastases [47].

Of course, the increased utilization of IAMs has improved the accessibility of patients, but it may also bring a greater economic burden to patients. Although most IAMs have a large price reduction under the background of the NDPN policy, the affordability of IAMs is still poor. Therefore, the government should formulate more specific policies to improve the accessibility of EAMs, and at the same time, should monitor the rapid growth of IAMs.

Finally, overall, there has been a great improvement in the affordability of EAMs and IAMs for urban and rural residents during this study. Although the burden capacity of urban and rural residents has increased compared to 2016, rural patients still cannot afford EAMs and IAMs; urban patients also bear a heavy economic burden when using EAMs (OBs) and IAMs.

In this study, the evaluation of the cost of anti-cancer medicines used by patients, excluding EAMs (LPGs), showed a general downward trend. However, most anti-cancer medicines are unaffordable for patients. 16 of the 17 IAMs in the study were included in the medical insurance Class-B catalog, and although 16 IAMs have different degrees of price reduction, the affordability of urban and rural residents has been significantly improved during the study period, but due to their expensive price, urban and rural residents still unaffordable. However, it was found that EAMs (LPGs) could reduce the treatment cost of cancer patients, and present relatively good affordability for both urban and rural residents.

It should be noted that cancer let patients passively accept and pay for anti-cancer medicines without considering the cost increase over other diseases for its life-threatening nature [48]. At the same time, high-cost anti-cancer medicines may lead to more families falling into poverty, causing more cancer patients to die prematurely due to a lack of drug treatment [49, 50]. Health policy should address the imbalance in the burden capacity of anti-cancer medicines between patients with different socioeconomic levels, especially for disequilibrium between urban and rural patients [51].

The government should adjust relevant policies and regulations, speed up the approval of EAMs (LPGs), and improve their market access [52]. Comprehensive coverage of EAMs may promote their use and reduce patients’ demand for expensive IAMs, especially when the two treatments produce similar clinical benefits. How to use health technology assessment, evidence-based medicine evidence, health economics evaluation, and other means to let patients get the maximum clinical benefit, and balance the pressure of medical insurance and enterprise development, is a huge challenge in the future.

### Limitations

First, this study was conducted in Nanjing, a relatively economically developed provincial capital city in eastern China, and cannot represent the trends and status of the entire province or even the country. Our research data comes from the procurement data of various hospitals, which cannot truly reflect the clinical use or acquisition situation but only reflects the overall demand trend of anti-cancer medicines over time. Second, although the defined daily dose (DDD) of anti-cancer medicines in this study has a reference source, it has not been verified by the World Health Organization (WHO), and affordability may be overestimated because medical costs outside of drugs have not been considered. Third, the study period was from 2016 to 2020, in China, the NEML implemented before November 1, 2018, was the 2012 version. 6 of the 24 essential medicines included in this study (including one targeted drug and two monoclonal antibodies) were not included in the 2012 NEML version. Therefore, the prices and utilization of essential medicines in 2019–2020 may be overestimated because three expensive varieties with innovative anti-cancer medicine properties were included.

## Conclusions

This study showed that the price, availability, drug utilization, and affordability of anti-cancer medicines in Nanjing have undergone positive changes. For anti-cancer medicines in medical institutions, the availability and drug utilization of IAMs showed an increasing trend, but EAMs still dominated cancer treatment. The results of this study demonstrate that some positive measures in medical reform have promoted a decrease in drug prices, especially for EAMs (OBs) and IAMs. Based on its national conditions, each country formulates corresponding medical policies, insurance policies, and drug policies. This study provides a true reflection of the actual situation in China and can serve as a basis for policy formulation by the Chinese government. The national level must formulate more effective policies to improve the availability of essential medicines. Although most families cannot afford IAMs, their affordability is increasing. At the same time, treating diseases and saving lives is a common goal worldwide. This article is intended for international exchange and mutual exchange of information.

### Electronic supplementary material

Below is the link to the electronic supplementary material.

Supplementary Material 1

## Data Availability

The original contributions presented in the study are included in the article, further inquiries can be directed to the corresponding author.

## Abbreviations

WHO: World Health Organization
EAMs: Essential anti-cancer medicines
IAMs: Innovative anti-cancer medicines
DDDc: Defined daily dose cost
DDDs: Defined daily doses
LPGs: Lowest-priced generics
OBs: Originator brands
NDPN: National drug price negotiation
IARC: International Agency for Research on Cancer
NEML: National essential medicines list
GPCAIAMs 2020: Guiding Principles of Clinical Application of Innovative anti-cancer Medicines
NHC: National Health Commission
IQR: Interquartile Range
DDD: Defined Daily Dose
LPGW: Lowest-paid unskilled government worker
NSCLC: Non-small cell lung cancer
