# Supplementary material for: Access to essential and innovative anti-cancer medicines: a longitudinal study in Nanjing, China
Source: BMC Health Serv Res. 2024 Jul 11;24:802. doi: 10.1186/s12913-024-11285-5 (PMC11242009; doi:10.1186/s12913-024-11285-5)
Supplement: Supplementary file 1 — Supplementary Material 1 [file 12913_2024_11285_MOESM1_ESM.docx]

**Table S1 Basic information of sample hospitals**

| **NO** | **Name of Medical Institution** | **Hospital Level** | **Patient’s age group** | **Area** | **Whether the hospital caters for all oncology diagnosis and treatments** |
| --- | --- | --- | --- | --- | --- |
| **1** | Nanjing Jiangning Hospital | Tertiary | Adults | Nanjing | YES |
| **2** | JiangSu Province Hospital | Tertiary | Adults | Nanjing | YES |
| **3** | The Second Affiliated Hospital of Nanjing Medical University | Tertiary | Adults/Children | Nanjing | YES |
| **4** | Nanjing Drum Tower Hospital | Tertiary | Adults | Nanjing | YES |
| **5** | Jiangsu Cancer Hospital | Tertiary | Adults/Children | Nanjing | YES |
| **6** | Jiangsu Province Hospital with Integration of Chinese and Western Medicine | Tertiary | Adults | Nanjing | YES |
| **7** | Nanjing First Hospital | Tertiary | Adults/Children | Nanjing | YES |
| **8** | Nanjing Chest Hospital | Tertiary | Adults | Nanjing | NO |
| **9** | Nanjing Tongren Hospital | Tertiary | Adults | Nanjing | YES |
| **10** | General Hospital Of East Theater Command | Tertiary | Adults/Children | Nanjing | YES |
| **11** | Zhongda Hospital Southeast University | Tertiary | Adults/Children | Nanjing | YES |
| **12** | Nanjing Municipal Government Hospital | secondary | Adults | Nanjing | YES |
| **13** | Nanjing Women and Children's Healthcare Hospital | Tertiary | Adults/Children | Nanjing | YES |
| **14** | Children's Hospital of Nanjing Medical University | Tertiary | Children | Nanjing | YES |
| **15** | The 81st Hospital of the People's Liberation Army | Tertiary | Adults | Nanjing | YES |
| **16** | Nanjing Gaochun Traditional Chinese Medicine Hospital | secondary | Adults | Nanjing | YES |
| **17** | Nanjing Xuanwu Hospital | secondary | Adults | Nanjing | YES |
| **18** | Nanjing Meishan Hospital | secondary | Adults | Nanjing | YES |
| **19** | Jiangsu Province Hospital of Chinese medicine | Tertiary | Adults/Children | Nanjing | YES |
| **20** | Nanjing Red Cross Hospital | secondary | Adults | Nanjing | YES |
| **21** | Nanjing Gaochun People's Hospital | secondary | Adults | Nanjing | YES |
| **22** | Jiangsu Province Official Hospital | Tertiary | Adults | Nanjing | YES |
| **23** | Nanjing Hospital of Chinese Medicine | Tertiary | Adults | Nanjing | YES |
| **24** | Nanjing Integrated Traditional Chinese And Western Medicine Hospital | Tertiary | Adults | Nanjing | YES |
| **25** | PLA Eastern Theater Command Air Force Hospital | Tertiary | Adults | Nanjing | YES |
| **26** | The Second Hospital of Nanjing | Tertiary | Adults | Nanjing | YES |

**Table S2 Relative changes in drug utilization of EAMs in 2016 and 2020**

| **Drug name** | **DDDs** **Relative changes of EAMs(LPGs), %** | **DDDs Relative changes of EAMs(OBs), %** |
| --- | --- | --- |
| Vincristine | -6.99 | NA |
| Trastuzumab | NA | 1491.09 |
| Tamoxifen | 4.14 | NA |
| Rituximab | NA | 194.97 |
| Paclitaxel | -23.30 | -26.42 |
| Oxaliplatin | 31.36 | 175.05 |
| olinic acid calcium salt hydrate | 23.15 | NA |
| Methotrexate | 52.66 | NA |
| Mesna | -100.00 | 21.23% |
| Mercaptopurine | 52.21 | NA |
| Imatinib | -97.33 | 99.26 |
| Ifosfamide | -94.19 | 156.19 |
| Hydroxyurea | 58.58 | NA |
| Gemcitabine | 45.42 | 165.35 |
| Fluorouracil | 32.66 | NA |
| Etoposide | 573.24 | NA |
| Daunorubicin | -89.94 | NA |
| Cytarabine | -51.97 | -5.93 |
| Cyclophosphamide | -99.86 | 233.27 |
| Cisplatin | 29.07 | NA |
| Carboplatin | 109.24 | 150.08 |
| Capecitabine | -71.37 | 246.92 |
| Asparaginase | -12.00 | NA |
| ArsenicTrioxide | -13.70 | NA |

**Table S3 Relative changes in drug utilization of IAMs in 2016 and 2020**

| **Drug name** | **DDDs Relative changes of IAMs,%** |
| --- | --- |
| Sunitinib | 22537.50 |
| Icotinib | 6728.24 |
| Apatinib | 3976.40 |
| Everolimus | 3580.00 |
| Lapatinib | 3233.33 |
| Sorafenib | 2651.35 |
| Bevacizumab | 1922.44 |
| Bortezomib | 1270.10 |
| Axitinib | 605.01 |
| Gefitinib | 591.61 |
| Erlotinib | 513.71 |
| Dasatinib | 471.76 |
| Rh-endostatin | 408.55 |
| Nilotinib | 400.00 |
| Chidamide | 135.77 |
| Nimotuzumab | 61.00 |
| Crizotinib | 10.22 |
